# Global mismatch between fishing dependency and larval supply from marine reserves

**DOI:** 10.1038/ncomms16039

**Published:** 2017-07-10

**Authors:** Marco Andrello, François Guilhaumon, Camille Albouy, Valeriano Parravicini, Joeri Scholtens, Philippe Verley, Manuel Barange, U. Rashid Sumaila, Stéphanie Manel, David Mouillot

**Affiliations:** 1 EPHE, PSL Research University, CEFE UMR 5175, CNRS, Université de Montpellier, Université Paul-Valéry Montpellier, Biogéographie et Ecologie des Vertébrés, 1919 route de Mende, 34293 Montpellier, France; 2 UMR 9190 MARBEC, IRD-CNRS-IFREMER-UM, Université de Montpellier, 34095 Montpellier, France; 3 Landscape Ecology, Institute of Terrestrial Ecosystems, ETH Zürich, 8092 Zürich, Switzerland; 4 Swiss Federal Research Institute WSL, 8903 Birmensdorf, Switzerland; 5 IFREMER, Unité Ecologie et Modèles pour l’Halieutique, 44300 Nantes Cedex 3, France; 6 CRIOBE, USR 3278 CNRS-EPHE-UPVD, Labex ‘Corail’, University of Perpignan, 66860 Perpignan, France; 7 MARE Centre for Maritime Research, Amsterdam Institute for Social Science Research, University of Amsterdam, Plantage Muidergracht 14, 1018 Amsterdam, The Netherlands; 8 IRD, UMR AMAP, TA A51/PS2, Montpellier, 05 34398 Cedex, France; 9 Plymouth Marine Laboratory, Prospect Place, PL1 3DH Plymouth, UK; 10 Food and Agriculture Organization of the UN, Viale delle Terme di Caracalla, 00153 Rome, Italy; 11 Fisheries Economics Research Unit, Institute for Oceans and Fisheries & Liu Institute for Global Studies, the University of British Columbia, Vancouver, Canada V6T 1Z; 12 Australian Research Council Centre of Excellence for Coral Reef Studies, James Cook University, Townsville, 4811 Queens Land, Australia

## Abstract

Marine reserves are viewed as flagship tools to protect exploited species and to contribute to the effective management of coastal fisheries. Yet, the extent to which marine reserves are globally interconnected and able to effectively seed areas, where fisheries are most critical for food and livelihood security is largely unknown. Using a hydrodynamic model of larval dispersal, we predict that most marine reserves are not interconnected by currents and that their potential benefits to fishing areas are presently limited, since countries with high dependency on coastal fisheries receive very little larval supply from marine reserves. This global mismatch could be reversed, however, by placing new marine reserves in areas sufficiently remote to minimize social and economic costs but sufficiently connected through sea currents to seed the most exploited fisheries and endangered ecosystems.

## Abstract (web summary)

The effectiveness of marine reserves relies on whether they supply substantial recruitment outside their boundaries. Here, Andrello and colleagues use models of larval fish dispersal to show that countries most dependent on coastal fisheries receive the lowest larval supplies from marine reserves.

Overexploitation of natural resources, climate change and other anthropogenic stressors are threatening the integrity of coastal marine ecosystems, their biodiversity and associated services^1^,^2^,^3^,^4^. In many coastal areas, fisheries constitute a primary source of food, income and labour and make large contributions to nation’s gross domestic product (GDP)^5^,^6^,^7^. Thus, the depletion of fish stocks may lead to social-ecological traps, where some dependent human communities increase resource use to alleviate poverty, with negative consequences for the state of the resource base^8^.

Marine protected areas (MPAs), and specifically no-take marine reserves (MRs), which are MPAs classified as strict nature reserves or wilderness areas^9^, are widely recognized as effective conservation tools supporting greater species biodiversity and biomass than nearby exploited areas^10^,^11^,^12^,^13^. MRs are also promoted as potential tools to assist the management of coastal fisheries by securing a portion of fish stocks and buffering fluctuations of fish populations facing overexploitation^14^. Moreover, by hosting abundant populations of exploited species, MRs could, through adult spillover and larval supply, provide net benefits to neighbouring areas and contribute to rebuilding overexploited fish stocks^15^,^16^,^17^,^18^,^19^.

While adult spillover is limited to few kilometres outside the reserve^15^,^16^, larval dispersal can reach up to hundreds of kilometres following prevalent sea currents^20^,^21^. For instance, larvae of coral reef groupers can disperse up to 200 km and effectively contribute to recruitment in distant exploited areas^21^. Larval connectivity can thus provide resilience to MPA networks against species loss, improving the effectiveness of MPAs and MRs networks for both biodiversity conservation and fisheries management support^22^,^23^, and deliver benefits to exploited areas at large spatial and temporal scales^15^,^21^. However, the extent to which the global system of MPAs is interconnected and able to seed areas where fisheries are the most critical for food and livelihood security is unknown. This is partly because of the inherent difficulty of tracking larval dispersal over long distances.

Larval dispersal can be estimated through various techniques with different strengths and weaknesses^24^. Parental genetic and otolith chemical analyses have been successfully employed to estimate larval dispersal between MPAs^20^,^21^. However, these methods require the sampling of a large number of individuals as well as costly and time-consuming analyses. Moreover, these methods are only effective when differences in genetics or otolith chemical elements are sufficiently contrasted between areas. As an alternative, biophysical dispersal models allow the indirect study of connectivity patterns at large spatial and temporal scales^25^,^26^. The main disadvantage of such model-based estimation is the dependence on model parameterization, among which the pelagic larval duration (PLD) has the largest effect for estimating large scale connectivity^27^. While empirical validation of these models remains challenging^28^,^29^, the effects of parameter uncertainty can be partly addressed using sensitivity analyses^30^.

Here, we use a hydrodynamic biophysical model to provide global-scale predictions of larval connectivity among MPAs and larval supply from MRs to areas open to fishing, particularly in regions with high economic and livelihood dependency on fisheries. We show that most MPAs are not interconnected and that the supply of larvae from MRs towards areas with high dependency on coastal fisheries is very limited. On the other hand, we reveal that strong oceanographic currents have the potential to deliver unexpected long-distance conservation benefits even to countries where MRs are currently absent. This result demonstrates that, beyond national conservation efforts and small-scale adult spill-overs, MRs can sustain transnational benefits, provided that their location is planned by explicitly considering marine connectivity patterns.

## Results

### Number and coverage of marine protected areas

The World Database of Protected Areas (WDPA, downloaded in June 2013)^31^ identifies a total of 3,061 coastal MPAs of which 695 (23%) are MRs with a total coverage of 0.9% of the world coastal areas (Supplementary Table 1). MPAs are considered marine reserves (MRs) if they satisfy at least one of the following criteria: they are fully no-take zones, they include a no-take zone or they are classified as strict nature reserves or wilderness areas (that is, IUCN categories Ia or Ib).

### Connectivity between marine protected areas

**Figure 1:**
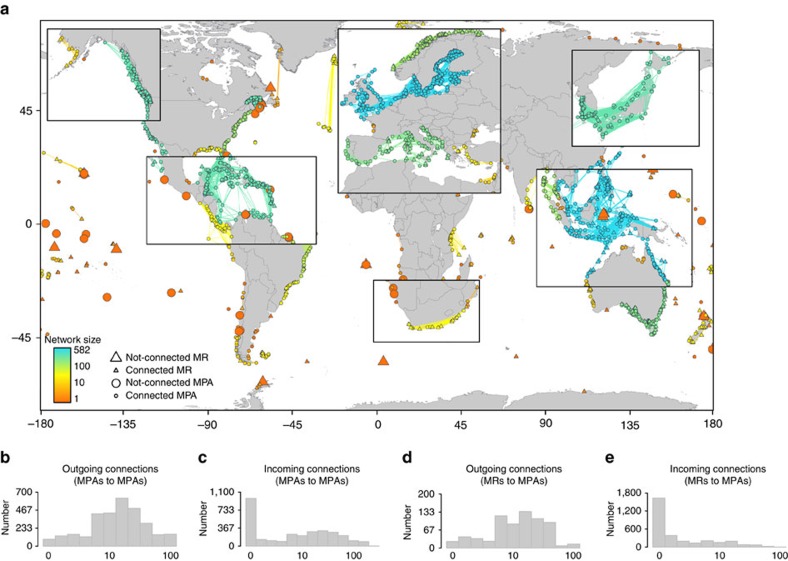
Global connectivity patterns among marine protected areas and no-take marine reserves based on larval dispersal patterns. (**a**) Regions with the largest networks of marine protected areas (MPAs, circles) and marine reserves (MRs, triangles) are enlarged for readability. Networks of connected MPAs and MRs are coloured according to their size. Unconnected MPAs and MRs are drawn with larger symbols. Histograms represent the distribution of the number of outgoing (**b**,**d**) and incoming (**c**,**e**) connections per MPA. In (**b**) and (**c**), all MPAs are considered as donors while in (**d**) and (**e**) only MRs are considered as donors.

To estimate the probability of larval dispersal between pairs of MPAs, we simulated the release of ten thousand virtual larvae from each MPA four times a year for 6 years. Larvae were let to drift passively over a period of 30 days, corresponding to the mean pelagic duration (PLD) of most fish species^32^. Connection probabilities are estimated by recording the position of each individual larva at the end of the PLD, and used to identify isolated and networked MPAs. These simulations indicate that MPAs are globally weakly connected, with only a few large networks that combine up to 582 MPAs in Northern Europe (Fig. 1a). The number of isolated MPAs is remarkably high: 969 MPAs (32%) are not seeded by any other MPA (zero incoming connections) and 61 (2%) are completely isolated (zero incoming and outgoing connections, larger symbols in Fig. 1a). On average, each MPA receives larvae from six other MPAs (interquartile range: 0–23) and sends larvae to 11 other MPAs (interquartile range: 5–21) (Fig. 1b,c). When only MRs are considered as donors, the number of isolated MPAs is even higher: 1,636 MPAs (53%) do not receive any larvae (Fig. 1d,e).

In addition to direct connections, we also quantified the connectivity within each network of MPAs using two metrics of centrality. The betweenness centrality (BC) identifies the MPAs acting as gateways of connectivity through multi-step connections, thus measuring their importance for multi-generational connectivity and gene flow^30^. The eigenvector centrality (EC), on the other hand, predicts the effects of catastrophic events, as it ranks single MPAs according to the reduction in metapopulation size that would result from their local extinctions^33^. The BC and EC of MPAs are generally not correlated (Spearman’s *ρ* between BC and EC <0.7 in 92% of networks), and are also uncorrelated with the number of connections (*ρ*<0.7 in 65% and 84% of networks for number of connections and BC and EC, respectively). Thus, central MPAs (with high BC and/or high EC) are not necessarily the ones with the highest numbers of connections. More importantly, central MPAs are not better protected than non-central ones since BC is not significantly different between MPAs and MRs (Wilcoxon rank sum test, *W*=770,810, *P*=0.95) while the ECs of MRs are lower than those of MPAs (one-sided Wilcoxon rank sum test, *W*=733,070, *P*=0.04).

As connections with low probabilities may be too weak to influence population dynamics across MPAs^34^,^35^, we assess the sensitivity of network connectivity using different thresholds in larval connection strength. When connectivity metrics are recalculated considering only connections above the first or the second tertile of the connectivity probability distribution, the number of networks and isolated MPAs is higher, but the median network size remains similar (Supplementary Fig. 1, Supplementary Table 2).

### Larval supply from marine reserves

**Figure 2:**
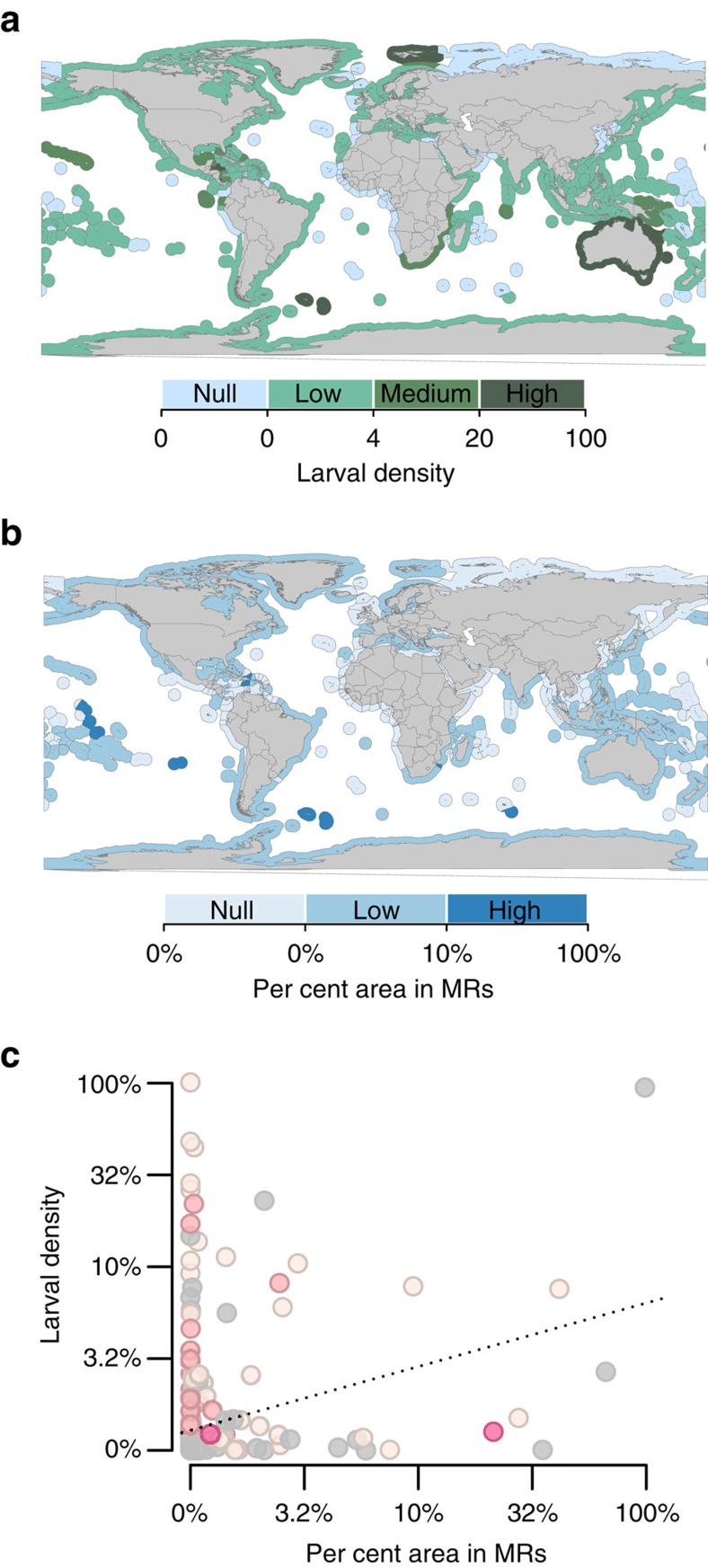
Larval supply from marine reserves to national exclusive economic zones. (**a**) Global map of larval density (unit-free index bounded between 0 and 100 reflecting the number of larvae received in a fishing area relative to the surface of the fishing area) in each country’s exclusive economic zone (EEZ), with darker green representing a higher density and lighter green representing lower or zero densities. (**b**) EEZs are coloured according to the per cent of coastal areas in MRs, with darker blue representing a higher percentage and lighter blue representing a zero percentage. (**c**) Relationship between larval density and per cent area of MRs in EEZs (*n*=289, *R*^2^=7%); dots are coloured according to the food security fisheries dependency in the country, with darker pink representing higher dependency and lighter pink representing lower dependency, as in Fig. 4c; countries without estimates of food security fisheries dependency are in grey.

Larval dispersal is not only a mechanism to strengthen networks of MPAs through a spatial insurance, but a potentially effective process to seed fishing areas and thus to provide benefits to coastal fisheries. We defined the coastal fishing area of each country as the portion of the coastal Exclusive Economic Zone (EEZ) open to fishing. To account for differences in fish biomass between MRs, which ultimately drives the amount of released larvae, we predicted fish biomass per unit of area in each MR using a statistical model fitted on a reduced number of MRs with known values^10^ and a set of environmental and socio-economic predictors (Supplementary Table 3 and Supplementary Data 1). Given the accuracy of the model (*R*^2^=80%), the predicted fish biomass in all MRs was used to weight the larval release potential of each MR and then to calculate the number of larvae dispersing to each EEZ. At the global scale, we observed that 37% of EEZs (*n*=109) do not receive larvae from MRs (Fig. 2a). Many African countries are in this category, particularly those bordering the Red Sea and the Eastern Mediterranean (Egypt and Sudan), along the West African coast, but also in South America (for example, Peru). Many isolated coastal areas are also unseeded, notably the Mascarene region, including Mauritius and La Réunion, in the Indian Ocean, the Azores and Cape Verde in the Atlantic Ocean, and many islands and archipelagos across the Pacific Ocean (for example, the Marshall Islands, Kiribati and Vanuatu). Conversely, the highest densities of larvae seeded from MRs are found in coastal areas of Australia, some remote islands (South Georgia and the South Sandwich Islands, Svalbard) and in the Caribbean (Belize, Costa Rica and Honduras).

**Figure 3:**
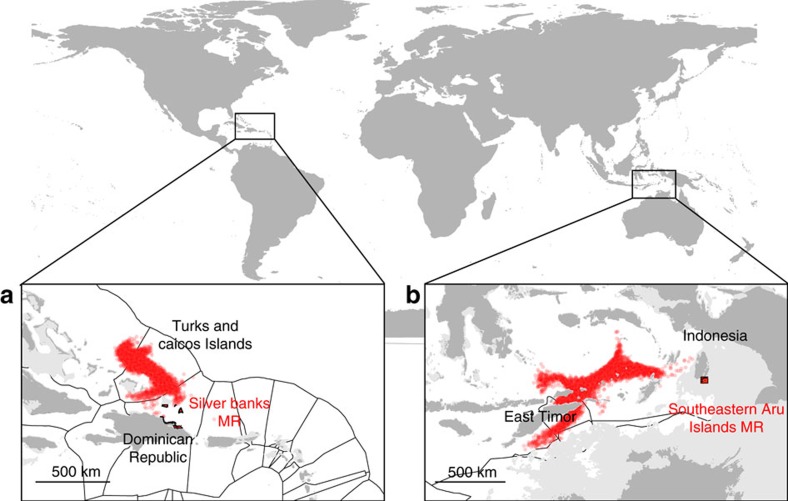
Examples of transnational larval supply from marine reserves to fishing areas. MRs providing larval supply to fishing areas in the Caribbean (**a**) and the Coral Triangle (**b**) regions. Land areas are coloured in dark grey, coastal areas in light grey, coastal areas in MRs in red and lines indicate EEZ boundaries. The red dots are the positions of fish larvae at the end of the larval dispersal phase (30 days).

Global patterns of larval supply from MRs are driven by several factors. First, the relative short pelagic larval duration (30 days) results in many larvae remaining within country boundaries; the median percentage of larvae recruiting in the EEZ where they originate is 86% (mean: 70%), compared to 14% (mean: 30%) dispersing to other EEZs (Supplementary Fig. 2 and Supplementary Data 2). Second, some countries have fewer and/or smaller MRs than others so a more limited seeding capacity. For example, 175 EEZs (61%) have no MRs (Fig. 2b). However, there is a weak relationship between the percentage of coastal area covered by MRs and larval supply at the EEZ level (Fig. 2c, linear regression on log-transformed values, *P*<0.001, *R*^2^=7%). Some EEZs receive very low larval densities despite considerable conservation efforts within their boundaries (for example, the Heard and McDonald Islands or the Line Group), whereas some EEZs with a very limited coastal surface area in MRs receive high densities of larvae (for example, Costa Rica). Indeed, for many EEZs, larval supply can be provided entirely by other countries (Supplementary Data 2), especially in regions where EEZs are small and clustered. For example, in the Caribbean, the Turks and Caicos Islands have no MRs but receive larvae from the Silver banks MR located in the Dominican Republic (Fig. 3a). In the Coral Triangle region, East Timor receives all its larval supply from MRs located in other countries, particularly Indonesia (Fig. 3b). Thus, beyond national conservation efforts and small-scale adult spill-over, oceanographic processes have the potential to expand the benefits of MRs through transnational source-sink dynamics.

### Fisheries dependency

**Figure 4:**
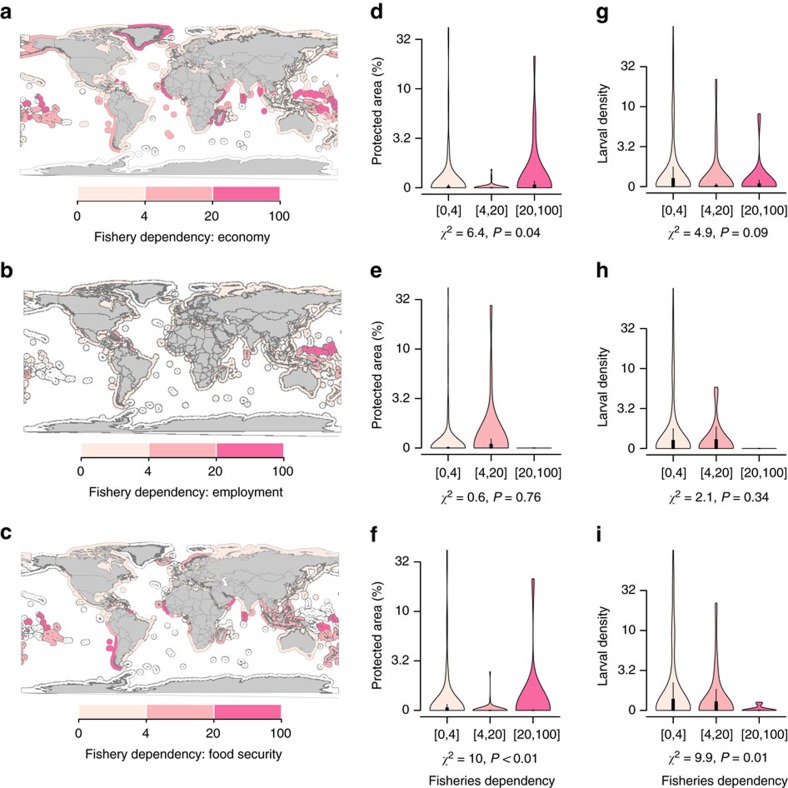
Relationships between national dependency on coastal fisheries and conservation effort or larval supply from marine reserves. The national dependency on coastal fisheries is calculated through unit-free indices bounded between 0 and 100 that estimate the economic (**a**,**d**,**g**, *n*=199), employment (**b**,**e**,**h**, *n*=144) and food security (**c**,**f**,**i**, *n*=139) dependency. Colours reflect the fisheries dependency, with darker pink representing higher dependency and lighter pink representing lower dependency. The violin plots in (**d**–**f**) compare the extent of protected coastal areas (measured as the percentage of EEZs in MRs) among countries grouped by the level of fisheries dependency. The violin plots in (**g**–**i**) compare the larval density from MRs among countries grouped by the level of fisheries dependency. The statistics under the plots show the results of a Kruskal–Wallis test. For both economic and food security dependency, the significant difference in area protected (**d**,**f**) is due to the difference between low and medium dependency classes (Conover post hoc test, *t*=2.6, *P*=0.03 and *t*=2.9, *P*=0.01, respectively). The significant difference in larval supply among food security dependency classes (**i**) is due to the differences between the high dependency class and the medium (*t*=2.4, *P*=0.02) and low (*t*=3.2, *P*<0.01) dependency classes.

For a given country, the relative importance of larval supply from MRs can be assessed in relation to the country’s dependency on coastal fisheries. The contribution of coastal fisheries to national welfare and wellbeing is multidimensional, with the provision of nutritious food, employment and economic value being most explicitly highlighted in the literature^1^,^5^,^36^,^37^. We quantify this through three indices of fisheries dependency using the economic value of coastal catches relative to the countries’ GDP (economic), the fraction of small-scale fishers out of the total active population (employment) and the catch per capita as a relative index of food security potential (food security)^1^. The countries with the highest dependency on coastal fisheries are located in West Africa (Guinea-Bissau, São Tomé and Príncipe, Senegal, Sierra Leone and Western Sahara) and in the equatorial Pacific (Kiribati, Micronesia, Solomon Islands and Tuvalu) (Fig. 4a–c). Other countries heavily dependent on coastal fisheries are Somalia, Turks and Caicos Islands, Andaman and Nicobar Islands, Maldives and Suriname.

Highly dependent countries have on average the same percentage of coastal area covered by MRs as less dependent countries (Fig. 4d–f; even if in absolute terms the surface area of MRs is larger for less dependent countries; Supplementary Table 4). However, as noted above, the percentage of coastal area covered by MRs is weakly correlated with larval supply owing to sea current patterns. Our dispersal model shows that larval supply from MRs is disproportionately concentrated in countries with low economic and nutritional dependency on coastal fisheries for their economy (Fig. 4g) and food security (Fig. 4i). Countries with a high nutritional dependency receive significantly less larvae than less dependent countries. Among the highly dependent countries, Guinea-Bissau, Kiribati, São Tomé and Príncipe, Senegal and Sierra Leone do not receive any larvae from MRs. Conversely, larval supply is not significantly different among countries with different levels of fisheries dependency in terms of employment (Fig. 4h).

### Uncertainty analysis

Our results can be affected by model parametrization and limitations, in particular larval behaviour (orientation and vertical migration) and PLD^27^. Due to a limited knowledge of larval behaviour, we considered larvae to be passive drifters, which can lead to an overestimation of dispersal distances^24^,^29^. We thus run another more conservative scenario where we decreased PLD from 30 to 20 days (corresponding to the first quartile of values for fishes^32^; Supplementary Table 2 and Supplementary Figs 2–5). Reducing the PLD has a marginal effect on the average number of connections or isolated MPAs, but as expected the larval supply to areas open to fishing decreases (higher number of unseeded EEZs), and countries with high fisheries dependence still receive significantly less larval supply from MRs.

## Discussion

Using a global hydrodynamic model of larval dispersal, we demonstrate that many MPAs (32%) are not connected and those that are the most important for network connectivity (high centrality) are not necessarily better protected (that is, no-take MRs) than less central MPAs. Connectivity confers resilience in case of local extinction, because larval supply from undisturbed sites can recolonize empty sites^38^. Thus, in addition to prioritizing new sites for protection on the basis of biodiversity needs^13^, spatial planning should ensure species persistence in case of potential local extinctions by increasing connectivity^39^. This requires the current system of MPAs to be complemented with new MPAs in zones that can cross-fertilize larvae with existing MPAs. For MPAs located in remote sites with small reef areas, for which external larval sources are minimal, the risk of catastrophic disturbance can be reduced by strengthening local protection through stronger restrictions and extensions of the protected area. These key features, when applying over long periods (>10 years), are known to efficiently promote fish abundance and diversity and increase mean fish body size^10^, which in turn improves the resilience of local populations to environmental disturbances^40^,^41^.

In addition to biodiversity conservation, MRs have the potential to provide benefits to neighbouring fisheries even at distances up to 200 km (refs 15, 21). We have shown that larval supply from MRs is unevenly distributed across fishing areas, with many EEZs (37%) not benefiting from any larval supply from MRs. In particular, many unseeded fished areas are located in countries with high fisheries dependency (for example, West Africa; Indo-Pacific region). Importantly, this is not a consequence of the absence of MRs in these countries^42^,^43^, but rather because sea surface hydrodynamics export larvae offshore or beyond the EEZ of the MRs.

The effects of ocean dynamics on the connectivity of MPAs show the importance of placing such areas strategically in response to conservation needs but also to the fishing needs of the most dependent communities. We argue that MRs should be created in areas with potential for larval transport towards fishing areas^44^,^45^ to ensure species protection, maximise provision of larval supply to neighbouring fisheries, while at the same time minimize the costs of protection. Encouraging results show that MRs could potentially help sustain small-scale fisheries in highly dependent countries^17^,^46^. The key to MR success in these cases is the collaborative partnership among local governments and their communities, demonstrating the potential benefits of MRs. This approach also presents MRs as a complementary tool to broader fisheries management frameworks, which increases effectiveness for both fisheries and biodiversity protection^14^,^47^.

An alternative to creating new MRs in areas with high social costs would also be to create new MRs in isolated or remote areas where conflicts are limited^48^. Such conservation efforts are rapidly expanding to fulfil at low cost the Aichi Biodiversity Target of 10% sea coverage but are coined as ‘residual’ reserves to extractive uses^48^,^49^. This is true inside MRs, given the limited net benefit of protection when human impact is almost absent^50^, but potentially wrong when expanding the benefits at larger scale given potential long-distance larval dispersal^21^,^51^. Another option is to place new MRs in the EEZs of countries with low fisheries dependency, from where larval dispersal through marine currents can seed critical fishing areas. Thus, adopting a transnational strategy to rebuild coastal fisheries through MRs would contribute to a more legitimate marine spatial planning, with more attention to the winners and losers of management intervention. This is even more important in the light of a recent study showing that individual reserves should export 30% or more of locally produced larvae to fishing grounds to sustain and rebuild adjacent fisheries and that 20–30% of fished habitats must be protected in 1–20 km wide reserves^52^. Unfortunately, such targets remain ambitious in many regions and alternative or complementary solutions must be found.

The importance of long-distance larval supply from MRs for fish population dynamics depends on mortality of fish larvae in open oceanic areas^24^ and on the intensity of exploitation and type of regulations in the seeded EEZ^14^,^18^, which will dictate whether incoming larvae constitute a significant and persistent contribution to recruitment. A few empirical studies support the benefits of larval export from MRs to adjacent fisheries^15^,^53^, but evidence of long-distance benefits is still scarce and in need of further work^21^,^51^. Another challenge is to show that the incoming species really contributes to biomass production and sustains fisheries. Depending on the country, some species contribute more to food security or economic incomes than others. However, larval supply could be relevant even if species are not consumed directly by humans. Sea currents will transport larvae of prey (fishes and invertebrates) and habitat builders, like on coral reefs, which altogether promote biodiversity and productivity of higher trophic levels that are consumed^41^. Given the high prevalence of diverse and non-selective fishing activities in countries highly dependent on fisheries, we consider that larval seeding from MRs could potentially benefit indirectly food security and human livelihoods.

Alternatively or in complement to larval supply from MRs, well-managed fisheries can be larval sources since (i) reductions in fishing effort can have larger benefits than the expansion of MRs^47^ and (ii) some areas where human populations and use of ecosystem resources is high can have surprisingly high fish biomass^54^. These ‘bright spots’ of management, able to be important sources of larvae while being fished, are not included in our model since (i) their extent is limited, (ii) their distribution highly patchy and (iii) their probability of occurrence challenging to predict at the global scale^54^. So, our study only focuses on larval supply by MRs where high fish biomass are consistently found while, for instance, more than a third of the fished reefs across the Indian Ocean show a biomass lower than 25% of that found in MPAs^55^.

In conclusion, the limited surface area protected in the sea and the unbalanced spatial design of MRs are failing to achieve the potential of current global conservation efforts to sustain human welfare and livelihoods. This global mismatch, given the target of 10% sea surface protected by 2020, could be reversed by the strategic establishment of new MRs in areas sufficiently remote to minimize social and economic costs and sufficiently connected through sea currents to seed the most critical fisheries and ecosystems.

## Methods

### Marine protected areas and marine reserves

The MPA database was downloaded from the World Database on Protected Areas^31^ in June 2013 and filtered before downloading to only keep protected areas known as ‘marine’. From this first set containing 9,600 MPAs, we retained only MPAs that passed the following sequential filtering criteria. First, we removed MPAs located completely on land using the land ecoregion of the world polygon (www.worldwildlife.org/biomes). Second, we removed MPAs covering pelagic area totally or for most of their surface using a depth limit of 200 m derived from the world bathymetric database ETOPO1 (ref. 56). Third, we deleted MPAs designated to protect species not considered in this study (for example, birds) by inspecting the ‘Designation’ field of the MPA shapefile. Fourth, in attempting to eliminate unreliable MPAs, we removed features with IUCN categories ‘Not recorded’ or ‘Not applicable’ (except national parks). The final database includes 3,061 MPAs of which 185 are classified as ‘fully no-take’ and 131 as ‘partly no-take’ (Supplementary Table 1). However, the fully and partly no-take designations do not coincide with the IUCN category Ia. We consider the 695 MPAs that are either in IUCN category Ia or Ib or designed at least partially no-take as effective MPAs (marine reserves, MRs). This criterion is less conservative than the one used in other studies finding a lower proportion of MRs, such as the one by Costello and Ballantine^9^ who considered only MPAs in IUCN category Ia as MRs (360 MPAs), concluding that only 6% of coastal and open sea MPAs are MRs.

### Exclusive economic zones

Exclusive economic zones (EEZs) are sea zones prescribed by the United Nations Convention on the Law of the Sea (1982) whereby a coastal State assumes jurisdiction over the exploration and exploitation of marine resources. EEZs extend from the coast to 200 nautical miles (370 km) off the coast. Polygons including inland waters, territorial waters and EEZs (that is, all areas where countries have exclusive rights and jurisdiction) were downloaded from the maritime boundary geodatabase^57^.

These polygons were intersected with the 12 marine biogeographic realms^58^ to obtain 289 biogeographic exclusive economic zones: these represent combination of country and biogeographic data, so that for example, Australia has two biogeographic exclusive economic zones (Central Indo-Pacific Australian region and the Temperate Australasia Australian region).

Coastal fishing areas are defined as the portion of biogeographic exclusive economic zones that are accessible by fishers since they are not protected: we removed the 695 polygons of marine reserves from the 289 biogeographic exclusive economic regions, using the ‘erase’ tool in ArcGIS 10.2. The resulting polygons, called EEZs in this study, thus represent the exclusive fishing areas of each country.

### Larval dispersal simulations

Sea surface current velocities are obtained by the Mercator Ocean’s Global ocean physical reanalysis GLORYS2V1 (ref. 59). The horizontal resolution of the model is 1/4° (∼28 km at the equator) and the temporal resolution of stored data is one day. The surface layer is one metre deep. The domain of the model is 180°W–180°E, 77°S–90°N. Data covered the period from January 1st 2003 to December 31st 2008, the most recent ones available at the time of this study.

Larval dispersal simulations were performed with Ichthyop 3.2 (ref. 60). Ten thousand virtual larvae were released in the centroid of each MPA at the midpoint of each season (that is, 2^nd^ February, 5^th^ May, 6^th^ August and 11^th^ November) in each of 6 years between 2003–2008, for a total of 734,640,000 released larvae. The time step of iteration was set to 3,600 s (1 h), which is sufficiently short for larvae not to cross more than one boundary of hydrodynamic cells in a single time step. Advection was simulated using a Runge-Kutta fourth order numerical scheme. Horizontal diffusion was applied by a random walk for individual larvae to account for sub-grid-scale hydrodynamics associated with coastal features (reefs, bays, gulfs, and so on) following Peliz *et al*.^61^, with a horizontal diffusion coefficient *K*=*ε*^1/3^*l*^4/3^, where *ε*=10^−9^ m^2^ s^−3^ is the constant turbulent dissipation rate^62^ and *l* is length of the grid cell.

Larvae were tracked for 30 days, corresponding to the mean PLD of fishes reported by Luiz *et al*.^32^. Using a single PLD value is in agreement with the most recent studies, which failed to find a latitudinal pattern in PLD^63^. Larvae were subject to passive dispersal only; this assumption has critical consequences on the dispersal distances of larvae and the patterns of connectivity, as active dispersal mechanisms such as swimming and orientation can increase local retention rates and decrease dispersal distances^27^. Integrating swimming larval traits, which are unknown for most species, would be prohibitive. To account for the effects of larval behaviour and to simultaneously evaluate the sensitivity of model results to the PLD, we run the hydrodynamics simulations using a PLD=20 days, as decreasing the PLD has similar effects to introducing some larval behaviour in the model. This is supported by results published in a previous study^30^, where we showed that simulating larval dispersal using vertical migration through current layers on 30 days is equivalent to dispersal on the top surface layers during 20 days.

### Connectivity among MPAs

We calculated the connection probabilities between all pairs of MPAs within the same marine biogeographic realm. The spatial position of larvae relative to MPA polygons was assessed using the function ‘gContains’ in the R package rgeos 0.3–19 on the latitude and longitude of each larva. Only the coastal portion of MPAs (shallower than 100 m, from ETOPO1) was considered suitable for the recruitment of larvae. Connection probabilities were used to construct a connectivity matrix among all MPAs.

Network metrics were used to characterize the global system of MPAs. In such a system, single MPAs are the nodes and connection probabilities are the edges of the network. Here, the word ‘network’ is used to denote a set of connected nodes (that is, a connected component), identified using the ‘components’ function of the R package igraph 1.0.1. Four network metrics were calculated for each node: the number of incoming edges, the number of outgoing edges, the betweenness centrality (BC) and the eigenvector centrality (EC). Since the global system of MPAs is composed of several independent networks, BC and EC were calculated separately for each network.

The number of incoming and outgoing edges were computed including connections of a node with itself using the ‘degree’ function of igraph. In the context of the present study, the number of incoming (respectively outgoing) edges measures the number of MPAs acting as donors (respectively receivers) of larvae for the focal MPA.

The BC of a node measures the importance of the node for the connectivity of the network. The BC of node *i* was calculated as the number of shortest paths between any two nodes that go through node *i*. The normalized betweenness centrality was calculated through the ‘betweenness’ function of igraph. In the context of the present study, the BC measures the importance of central MPAs for multi-step, multi-generational connectivity, which take advantage of single MPAs acting as central nodes to spread genes and individuals between MPAs that are not directly connected^30^.

The EC measures the influence of a node in a network. It is calculated as the left eigenvector associated with the leading eigenvalue of the connectivity matrix. Eigenvalues and eigenvectors were calculated using the ‘eigen’ function in R and eigenvectors were normalized between 0 and 1 to provide a ranking of nodes. In the context of the present study, a network of MPAs can be thought of as a network of local demes constituting a metapopulation. It has been shown that the EC of a node is proportional to the reduction in metapopulation size that would result from the removal of that node from the network^33^. The EC therefore measures the consequences of random catastrophic events leading to the extinction of local demes located in MPAs.

Theoretical studies show that external larval supply can regulate the demography of a local population if the number of supplied individuals is larger than the number of individuals removed from the local population (by death or emigration)^34^,^35^. If the number of incoming larvae cannot offset the number of deaths and emigrants, then larval supply has no positive effects on local demography. Therefore, a precise assessment of persistence in a set of connected populations requires quantifying both the number of incoming larvae (dependent on connection probabilities and source strengths) and local mortality (dependent on local demography), but this is beyond the scope of this study. Nevertheless, it is likely that the weakest connections do not deliver a sufficient number of larvae to the receiving MPAs. We thus provided all estimates of connectivity using (i) all connections, (ii) medium and strong connections only (second and third tertiles of connection probabilities) and (iii) strong connections only (third tertile). This analysis allows us to assess the robustness of our results to the removal of the weakest connections (those that might not deliver a sufficient number of immigrants).

### Fish biomass per unit of area in marine reserves

To predict fish biomass per unit area for the 695 MRs, a relationship was established between fish biomass estimates per unit area obtained from field surveys and a set of 12 environmental and socio-economic variables (Supplementary Table 3). Estimates of fish biomass per unit area come from Edgar *et al*.^10^ for a set of 121 MPAs worldwide. The environmental and socio-economic variables were collected from public databases^64^,^65^. The index of population pressure was calculated by fitting a quadratic kernel density surface (‘heatmap’ plugin in QGis) to each settlement point on a year 2000 world population density grid^66^.

The relationship between the log_10_ of fish biomass per unit area and the 12 predictors was modelled through a boosted regression tree (BRT) with an explanatory power of *R*^2^=80% (Pearson correlation between observed and predicted values *r*=0.90, *t*=22.46, d.f.=119, *P*<2.2 × 10^−16^). The relative influence of the predictors is listed in Supplementary Table 3. A simplified model retaining only the first eight variables was used to predict fish biomass per unit area for the 695 MRs of this study (Supplementary Data 1). The BRT analysis was performed using the functions ‘gbm.step’, ‘gbm.simplify’ and ‘gbm.predict’ of the R packages gbm 2.1.1 and dismo 1.1-1.

### Larval supply from marine reserves

We calculated the connection probabilities from the 695 MRs to the 289 EEZs of the same marine biogeographic realm. The spatial position of larvae relative to EEZ polygons was assessed using the function ‘gContains’ in the R package ‘rgeos’ on the latitude and longitude of each larva. Only the coastal portion of EEZs (shallower than 100 m, from ETOPO1) was considered suitable for the recruitment of larvae. Larval density (*LD*_*i*_) for a given EEZ was calculated as:




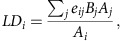




where *e*_*ij*_ is the fraction of larvae released in MR *j* that seed EEZ *i*, *B*_*j*_ is the fish biomass per unit of area in MR *j*, *A*_*j*_ is the coastal surface area (areas shallower than 100 m, from ETOPO1) of MR *j* and *A*_*i*_ is the coastal surface area of EEZ *i*. The product *B*_*j*_*A*_*j*_ is the predicted fish biomass in MR *j* and was used to account for differential larval production among MRs. The *LD*_*i*_ values were indexed by subtracting the minimum from the raw values and dividing them by the maximum–minimum deviation and multiplying them by 100, so as to obtain values bounded between 0 and 100.

Differences in *LD*_*i*_ among countries grouped by their level of fishery dependency were tested using a Kruskal–Wallis test. Post hoc differences between pairs of groups were tested using a Conover test; *P* values are adjusted using the Benjamini–Hochberg method. The analyses are conducted with the R package PMCMR 4.1.

### Fisheries dependency

The fisheries dependency assesses the importance of a coastal country’s small-scale fisheries in terms of their contributions to a national economy, employment and food security. These are the socio-economic indices that are the most explicitly highlighted in the literature^1^,^5^,^36^,^37^. While additional dimensions like recreation and tourism are important too^67^, we lack quantitative assessment at the global scale and evidence that these dimensions are directly related to larval seeding. We thus quantify fishery dependency following the methodology proposed and applied by Barange *et al*.^1^ and building on data obtained from the Sea Around Us project^68^. We focused only on coastal small-scale fisheries, because fisheries outside EEZs and in the high-seas are less likely to benefit from coastal MRs.

The economic indicator was calculated as the ratio of landed value of small-scale marine fishing (artisanal and subsistence fishing in the Sea Around Us database) to the country’s gross domestic product. We average these data over five years and use the most recent available values (2006–2010 in the Sea Around Us database).

The employment indicator was calculated as the ratio of the number of small-scale marine fishers to the national economically active population. Contributions were obtained from statistics of the United Nations Food and Agriculture Organization and International Labour Organization and from published literature^5^,^69^,^70^. Data on small-scale fisheries provided by member countries typically lead to an underestimation of employment^5^. The extrapolations made by Teh and Sumaila^5^ compensate for the underestimation of small-scale fisheries, but occasionally appear to overestimate employment^1^. Therefore, we took the average value of the extrapolated data of Teh and Sumaila^5^ and the reported data from available country-level studies^69^,^70^.

The food security indicator was calculated as the ratio of the biomass of small-scale marine fishing per capita (using the 2006–2010 average values form the Sea Around Us) scaled to an indicator of national diet adequacy. The indicator of diet adequacy is the ratio of national average animal protein intake per capita per day to the required level of 36 g per capita per day^1^. In this way, a country is considered highly dependent on marine fisheries in terms of food security if fish consumption (derived from artisanal and subsistence fisheries) per capita is high and if total animal protein consumption is low compared to a reference point (indicating an overall inadequate diet).

The three indicators were indexed by subtracting the minimum from the raw values and dividing them by the maximum–minimum deviation, and multiplying them by 100, so as to obtain values bounded between 0 and 100.

The implicit assumption of the analysis of larval supply in relation to fisheries dependency is that the extra influx of larvae can directly or indirectly contribute to the fisheries. While some species may contribute more to food security and others to economy, all larvae can affect the dynamics of fished ecosystem and thus contribute to local fisheries. Thus, larval supply can be relevant even if species are not consumed directly by the human communities.

### Data availability

The data that support the findings of this study are available from the corresponding author upon reasonable request.

## Additional information

**How to cite this article:** Andrello, M. *et al*. Global mismatch between fishing dependency and larval supply from marine reserves. *Nat. Commun.* **8,** 16039 doi: 10.1038/ncomms16039 (2017).

**Publisher’s note:** Springer Nature remains neutral with regard to jurisdictional claims in published maps and institutional affiliations.

## Supplementary Material

Supplementary Information

Supplementary Data 1

Supplementary Data 2
