# Supplementary material for: Global mismatch between fishing dependency and larval supply from marine reserves
Source: Nat Commun. 2017 Jul 10;8:16039. doi: 10.1038/ncomms16039 (PMC5508853; doi:10.1038/ncomms16039)
Supplement: Supplementary Information [file ncomms16039-s1.pdf]

Type of file: PDF
Title of file for HTML: Supplementary Information
Description: Supplementary Figures and Supplementary Tables.

Type of file: XLSX
Title of file for HTML: Supplementary Data 1
Description: List of the 695 marine reserves (MRs) considered in this study, with the geographic coordinates of the larval release point and the biomass per unit or area estimated with the boosted regression tree analysis.

Type of file: XLSX
Title of file for HTML: Supplementary Data 2
Description: List of the 289 exclusive economic zones (EEZs) considered in this study (see methods) with the total coastal surface area, the total area in marine reserves (MRs), the fisheries dependency indices and the larval density. Local retention is the fraction of larval supply originating from the MRs of the EEZ out of the total larvae produced by the MRs of the EEZ. Self-recruitment is the fraction of larval supply originating from the MRs of the EEZ out of the total larval supply to the EEZ. For EEZs with zero larval supply, these fractions are not calculated (NA).

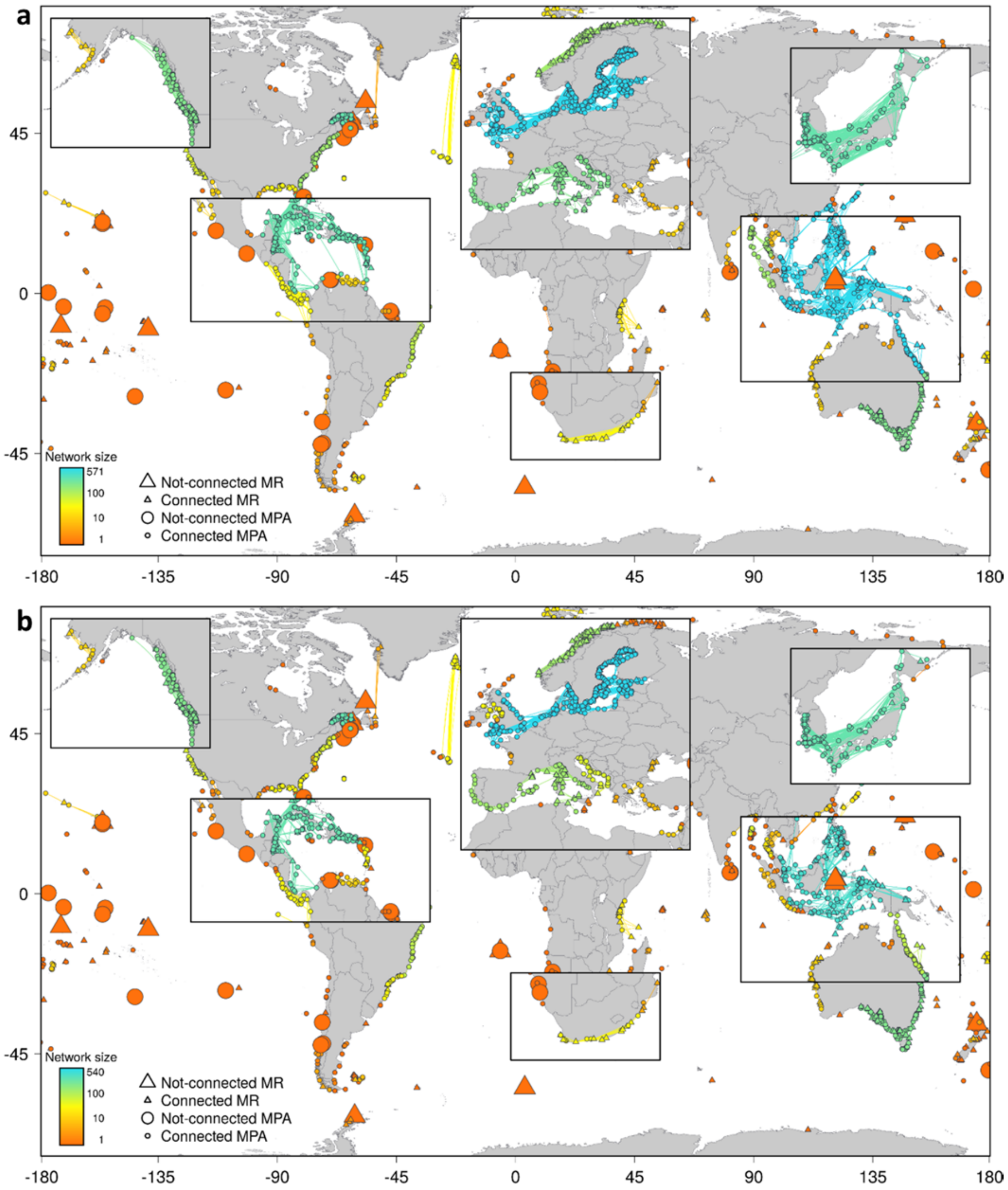


**Supplementary Figure. 1.**
Global connectivity patterns among MPAs (circles) and MRs (triangles) considering medium and strong connections only (second and third tertiles of connection probabilities, a) or strong connections only (third tertile, b). Regions with high MPA density are enlarged for readability. Networks of connected MPAs are coloured according to their size. Not-connected MPAs and MRs are drawn with larger symbols. Compare with Fig. 1A, depicting the case considering all connections.

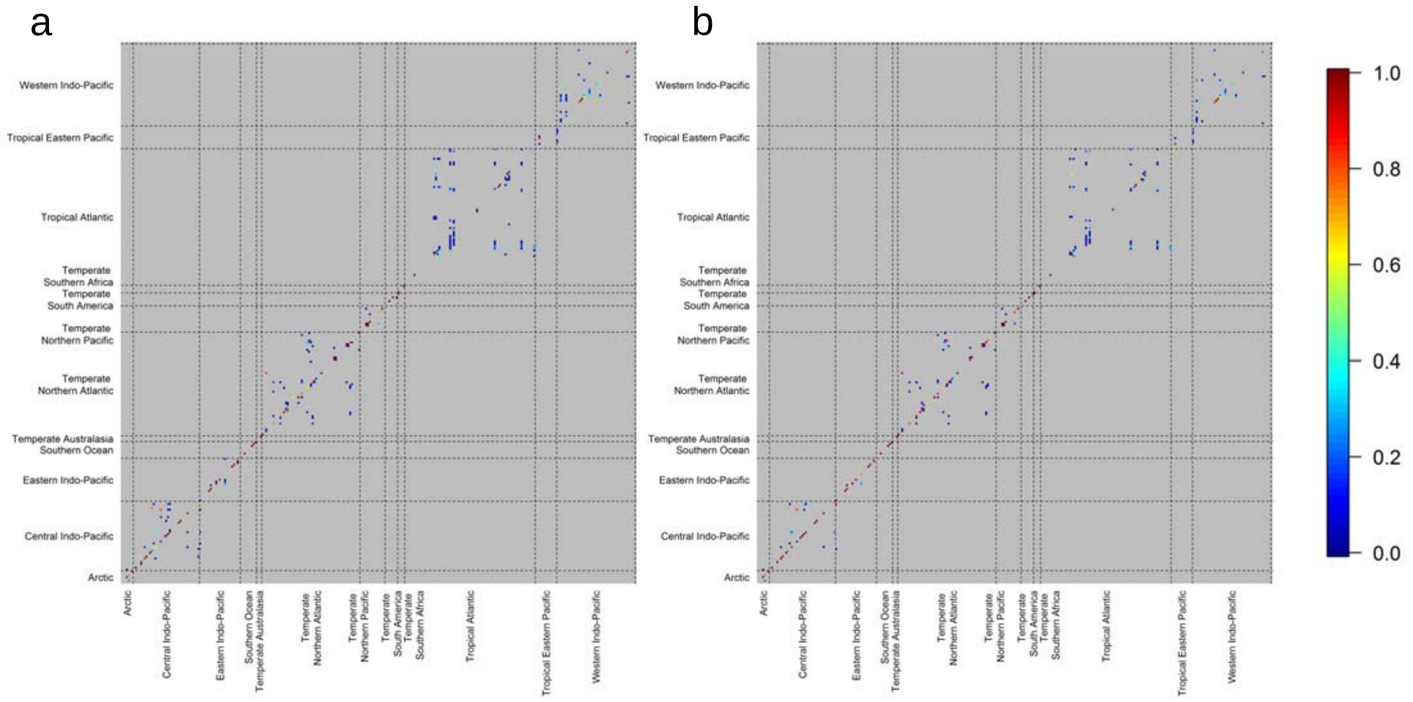


**Supplementary Figure 2.**
Connectivity matrices between MRs and coastal fishing areas for PLD = 30 days (**a**) and 20 days (**b**). Larval supply originating from MRs and directed to the coastal fishing areas for each pair of countries (*n* = 289), grouped by biogeographic realm. Colors represent the probabilities that a recruiting larva (i.e. not counting larvae lost in unfavorable habitats) originating from a MR of country *j* (column) is transported to the EEZ of country *i* (line). The diagonal thus represents the probabilities that larvae produced in a country's MRs will supply the coastal fishing areas of the same country.

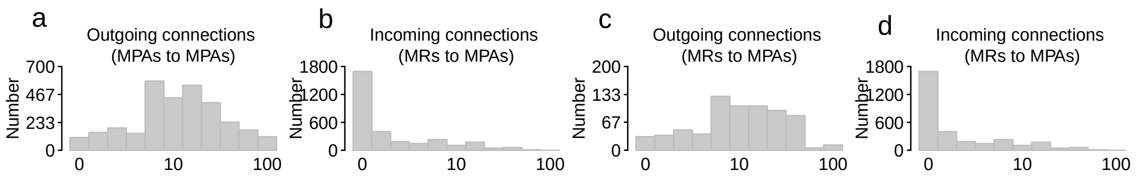


**Supplementary Figure 3.**
Connectivity among MPAs and MRs for PLD = 20 days. Histograms represent the distribution of the number of outgoing (**a** and **c**) and incoming (**b** and **d**) connections per MPA. In **a** and **b**, all MPAs are considered as donors while in **c** and **d** only MRs are considered as donors. Compare with Fig. 1B-E, depicting the case of PLD = 30 days.

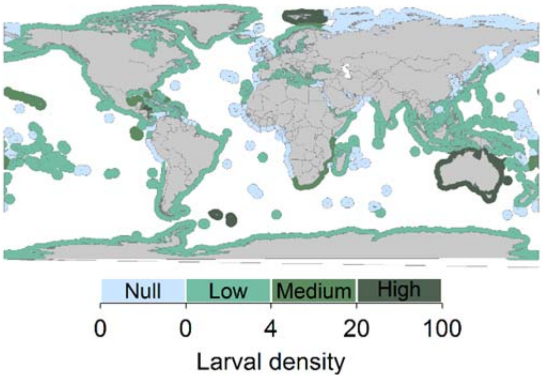


**Supplementary Figure 4.**

Larval supply from MRs to national EEZs for PLD = 20 days. Global map of larval density (unit-free index bounded between 0 and 100 reflecting the number of larvae received in a fishing area relative to the surface of the fishing area) in each country's exclusive economic zone (EEZ), with darker green representing a higher density and lighter green representing lower or zero densities. Compare with Fig. 2A, depicting the case of PLD = 30 days.

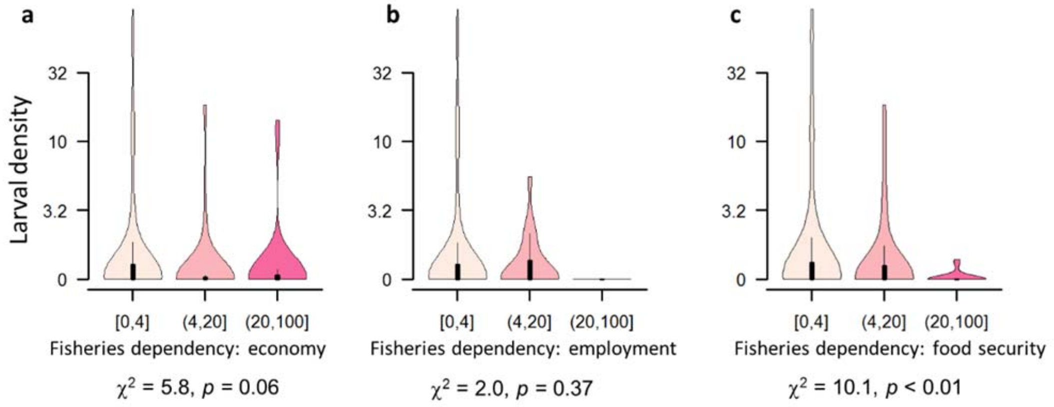


**Supplementary Figure 5.**
Differences in larval density from MRs among countries grouped by the level of fisheries dependency. Colours reflect the fisheries dependency (unit-free indices bounded between 0 and 100), with darker pink representing higher dependency and lighter pink representing lower dependency. The statistics under the plots show the results of a Kruskal-Wallis test. For food security dependency (**c**), the significance difference in larval supply is due to the difference between the high dependency class and the medium (Conover post-hoc test $t = 2.4$, $p = 0.03$) and low ($t = 3.2$, $p < 0.01$) dependency classes. Compare with Fig. 4g, h and i, depicting the case of PLD = 30 days.

**Supplementary Table 1**

Number of MPAs in each IUCN and fishing restriction category. IUCN categories classify protected areas according to their management objectives, going from 'strict nature reserves' (Ia) and 'wilderness areas' (Ib) to 'protected areas with sustainable use of natural resources' (VI) (https://www.iucn.org/theme/protected-areas/about/protected-areas-categories).

| IUCN category | Fishing restriction category | | | *Total* |
|---|---|---|---|---|
| | Accessible | Partly no-take | Entirely no-take | |
| Ia | 325 | 6 | 29 | *360* |
| Ib | 54 | 1 | 1 | *56* |
| II | 422 | 28 | 36 | *486* |
| III | 70 | 0 | 9 | *79* |
| IV | 779 | 57 | 30 | *866* |
| V | 588 | 4 | 25 | *617* |
| VI | 502 | 35 | 55 | *592* |
| Not Applicable | 5 | 0 | 0 | *5* |
| *Total* | *2745* | *131* | *185* | *3061* |

**Supplementary Table 2**

Effect of connection strength (all connections, medium and strong connections only, strong connections only) and PLD (30 days or 20 days) on the global connectivity patterns among MPAs and MRs. Medium and strong connections correspond to the second and third tertiles of connection probabilities; strong connections correspond to the third tertile.

| Connection strength | All | Medium and strong | Strong | All | Medium and strong | Strong |
|---|---|---|---|---|---|---|
| PLD | 30 | 30 | 30 | 20 | 20 | 20 |
| Connectivity between MPAs and MRs | | | | | | |
| Number of networks | 185 | 284 | 467 | 228 | 322 | 587 |
| Network size[a] | 5 (2 - 18) | 4.5 (2 - 12.7) | 5 (2 - 16.2) | 5 (2 - 13) | 4 (2 - 12) | 4 (2 - 12.2) |
| Number of incoming connections[a] | 6 (0 - 23) | 2 (0 - 14) | 0 (0 - 5) | 6 (0 - 19) | 2 (0 - 12) | 0 (0 - 5) |
| Number of outgoing connections[a] | 11 (5 - 21) | 7 (3 - 14) | 2 (4 - 7) | 9 (4 - 18) | 6 (3 - 12) | 3 (1 - 6) |
| Number of MPAs with no outgoing connections | 90 (2.9%) | 174 (5.7%) | 352 (11.5%) | 106 (3.5%) | 176 (5.7%) | 414 (13.5%) |
| Number of MPAs with no incoming connections | 969 (31.7%) | 1390 (45.4%) | 1883 (61.5%) | 934 (30.5%) | 1342 (43.8%) | 1812 (59.2%) |
| Number of MPA with no incoming connections from MRs | 1636 (53.4%) | 1963 (64.1%) | 2347 (76.7%) | 1694 (55.3%) | 1999 (65.3%) | 2352 (76.8%) |
| Larval supply to fishing areas[b] | | | | | | |
| Number of unseeded EEZs | 109 (37.9%) | | | 123 (42.7%) | | |
| Percentage of larvae recruiting in the EEZ of production[a] | 86% (44% - 100%) | | | 95% (58% - 100%) | | |
| Percentage of larvae dispersing to other EEZs[a] | 14% (0% - 56%) | | | 5% (0% - 42%) | | |

[a] Median (25% and 75% percentile)

[b] Metrics of larval supply are calculated with all connections because they take into account the connection strength

**Supplementary Table 3**

Predictors of fish biomass per unit of area in the 117 MPAs studied by Edgar et al. (2014). The last column lists the relative influence of each variable in a BRT analysis. The first eight variables with the largest influence (in bold) are retained after a simplification of the model and are used to predict fish biomass per unit of area for the 695 MRs of this study.

| Variable | Unit | Relative influence |
|---|---|---|
| Mean photosynthetically active radiation | Einstein $m^{-2}$ $day^{-1}$ | **29.5** |
| Sea surface temperature range | °C | **14.2** |
| Mean chlorophyll A concentration | mg $m^{-3}$ | **11.1** |
| Population pressure | Index | **10.2** |
| Mean sea surface temperature | °C | **9.9** |
| Mean nitrate concentration | µmol/l | **8.2** |
| Mean silicate concentration | µmol/l | **7.3** |
| Mean phosphate concentration | µmol/l | **6.0** |
| Gross domestic product | US$ | 1.3 |
| Government effectiveness | Index | 1.3 |
| Control for corruption | Index | 0.8 |
| Oceanic island isolated from continental shelf | Yes/No | 0.2 |

**Supplementary Table 4**

Total surface coastal area in MRs (km$^2$) by each fisheries dependency category.

| Fishery dependency | Low [0,4] | Medium (4,20] | High (20,100] | Total |
|---|---|---|---|---|
| Economy | 22 497 (86%) | 1 736 (7%) | 1 973 (7%) | 26 207 (100%) |
| Employment | 21 174 (85%) | 3 656 (15%) | 0 (0%) | 24 830 (100%) |
| Food security | 22 128 (90%) | 1 398 (6%) | 1 001 (4%) | 24 527 (100%) |
